# Correlates of Overweight and Obesity in German Primary School Children

**DOI:** 10.3390/nu16233987

**Published:** 2024-11-21

**Authors:** Anna Reißner, Olivia Wartha, Jens Dreyhaupt, Susanne Kobel

**Affiliations:** 1 Division of Sports and Rehabilitation Medicine, Ulm University Medical Centre, 89073 Ulm, Germany; 2 Institute for Epidemiology and Medical Biometry, Ulm University, 89075 Ulm, Germany

**Keywords:** obesity, children, health behaviour, lifestyle, chronic disease

## Abstract

Background/Objectives: Childhood obesity is a consistent public health issue, which often persists into adulthood. This study determined risk factors of childhood obesity and associated comorbidities in German school children. Methods: Data of 1956 primary school children (7.1 ± 0.6 years) were analysed. Anthropometrics were taken on site, other (health) parameters were assessed using a parental questionnaire. Binary logistic regression models were calculated, controlling for age, gender, family education level, and migration background. Results: Father’s physical illness (OR 1.092 [1.177; 3.073], *p* = 0.009) and a child’s chronic illness (OR 1.687 [1.077; 2.644], *p* = 0.022), maternal and paternal overweight (OR 2.180 [1.492; 3.185]; OR 2.494 [1.547; 4.022], *p* < 0.001, respectively), as well as maternal and paternal smoking (OR 1.942 [1.306; 2.889]; OR 1.972 [1.334; 2.917], *p* = 0.001, respectively) were significantly associated with the child being overweight. Also associated with the child being overweight were physical activity, regular screen media use (OR 0.605 [0.408, 0.896], *p* = 0.012; OR 2.029 [1.306; 3.152], *p* = 0.002, respectively), the mother and/or father thinking their child is too fat (OR 2.213 [1.504; 3.258], *p* < 0.001; OR 1.537 [1.048; 2.253], *p* = 0.028), the father’s physical inactivity (OR 1.69 [1.133; 2.521], *p* = 0.010), and if the child has not been breastfed (OR 1.632 [1.056; 2.521], *p* = 0.027), the mother smoked during pregnancy (OR 1.992 [1.224; 3.246], *p* = 0.006) as well as if the mother and/or father admonished their child about their weight (OR 25.521 [14.578; 44.680]; OR 19.448 [11.865; 31.877], *p* < 0.001, respectively). Children of unemployed mothers and in low-income households had an increased risk of being overweight (OR 4.811 [1.642; 14.096], *p* = 0.004; OR 2.203 [1.360; 3.568], *p* = 0.001, respectively). Conclusions: This study shows that parental health, lifestyle, and social behaviours matter in determining childhood obesity. Understanding those is essential to promoting a healthy lifestyle.

## 1. Introduction

Overweight and obesity have been a public health issue for several decades now [1,2]. This pandemic is not only affecting adults, but also more and more children [3]. In Germany, approximately 15% of all children are overweight and/or obese [4], which increased by 50% since 1990 and has stagnated on this high plateau since 2003 [4,5,6].

Comorbidities of obesity in childhood are both physical and psychological. Thus, the complex development of childhood obesity and its comorbidities across the lifespan should be understood through their environmental, psychosocial, and behavioural physiological determinants, which may involve the children, parents, and the society.

Obesity in childhood and adolescence can not only be associated with physical comorbidities but also with poor overall quality of life, low self-esteem, susceptibility to behavioural problems, poorer peer relationships, and higher rates of depression [7]. Further, it has been shown that overweight children are often confronted with bullying and stigmatisation [7].

It has been shown that children who are overweight early in life often continue to struggle with being overweight in adulthood [8]. Physical and psychological stressors, such as coronary heart disease [9], type 2 diabetes mellitus [10], cancer [11,12], and depression [13], which are related to obesity, can therefore occur in adulthood. It has been shown that obesity is associated with both increased mortality [1] and increased mortality rates, such as those for cancer [14] or cardiovascular events [15].

Risk factors for childhood obesity are diverse. There are genetic factors including chronic diseases such as asthma [16], diabetes mellitus [17], or attention deficit hyperactivity disorder [18]. Other genetic factors can also increase the risk of childhood obesity. If the child was born with an increased weight [19], before the 37th week of pregnancy [20], or if the mother had gestational diabetes during pregnancy [21], these can be risk factors for developing obesity in childhood. Further, maternal obesity can be associated with an increased risk and a child’s birth weight being above the 90th percentile [22,23]. However, parental obesity can also be associated with child obesity independently of the factors mentioned [24]. In overweight parents, both hereditary factors [25,26] and epigenetic modulations of the child’s metabolism [27] as well as the parenting style [28,29] and the parental role as role models are associated with an increased risk of obesity in childhood [30].

Parents can influence children’s diet by offering the right quantity and quality of food. It was shown that in addition to food quality [31] and quantity [32,33], eating behaviours, such as breakfast skipping, and the consumption of sugar-sweetened beverages can play a role in the development of obesity [34,35]. In addition, an excessive energy intake and insufficient energy expenditure is a reason for overweight. A child’s physical activity, on the other hand, can have a protective effect on the development of child obesity [36,37]. This can include parental physical activity [38,39], supporting the child’s physical activity [38,40], and joint parent–child activities [41]. Then again, increased media consumption is associated with lower physical activity [42]. Yet, other parental behaviours, such as cigarette smoking, are also associated with childhood obesity [43]. Still, there are also non-modifiable factors associated with childhood obesity, such as a low socio-economic status, single-parenthood, and a mother’s young age, which are all associated with an increased risk of childhood obesity [4,44,45,46].

So far, some risk factors for childhood obesity have been identified. However, only a few studies have been conducted in Germany [47,48,49] and are therefore only partly applicable to children living in Germany. Most factors identified were of socio-economic and demographic origin [47,48]. But there are—amongst others—also aspects such as nutritional and physical activity behaviour, sleeping patterns, genetics, and diseases. However, those aspects were detected in nation-wide studies including children and adolescents, with no distinction between young children and older adolescents. Further, the demographics in southwest Germany differ from those of many other states in Germany, especially with regard to migration, rural areas, and employment. Identifying which risk factors are primarily relevant at an early primary school age is important to take adequate preventive measures at an early age. Some of those preventive measures succeeded, resulting in children spending less time with screen media [50] and knowing about stress management and healthy eating [51]. It has been shown that preventive interventions have the potential to lead to children consuming more healthy foods and less junk food [52] or sweetened drinks [53], and therefore reducing their BMI [52]. Nevertheless, there are also some studies in which no significant effect could be achieved through prevention [54,55]. Therefore, a clear presentation of risk factors for overweight and/or obesity in children is necessary to find starting points for prevention and early intervention.

The aim of this work is to present risk factors for overweight and/or obesity in children transparently to create a basis for the prevention and early intervention of childhood overweight. By presenting risk factors, there is a chance that they can be neutralised or eliminated in a more targeted and precise manner through programmes and interventions.

## 2. Materials and Methods

### 2.1. Participants

Baseline measurements of 1956 primary school children (7.1 ± 0.6 years; 51.1% male) who participated in the school-based health-promotion programme “Join the Healthy Boat” [56] in southwest Germany were used for analysis. After schools (deans and teachers) agreed to take part, the written, informed consent of the parents as well as child assent were obtained prior to data collection. The study was approved by the Ministry of Culture and Education as well as the University’s ethics committee (application no. 126/10) and is in accordance with the declaration of Helsinki.

### 2.2. Anthropometric Measures

Children’s height (cm) and body mass (kg) were taken by trained technicians during a school visit according to standard procedures (in a vest, shorts, and with bare feet; Malina 1995). Standing height was measured to the nearest 0.1 cm using a stadiometer (Seca 213, Seca Weighing and Measuring Systems, Hamburg, Germany) and body mass was measured with electronic scales (Seca 862, Seca Weighing and Measuring Systems, Hamburg, Germany) to the nearest 0.05 kg. Subsequently, body mass index (BMI) was calculated as kg/m^2^ and converted to BMI percentiles (BMIPCT) using German reference data [57]. Overweight and obesity were determined above the 90th and 97th percentile (BMIPCT), respectively.

Parental weight status (BMI) was calculated based on self-reported height and weight. A BMI ≥ 25 was classed as overweight (including obesity).

### 2.3. Risk Factors and Correlates

Potential risk factors and behavioural correlates derived from parental responses (based on the KiGGS survey, which assessed health behaviour in 18,000 German children and adolescents [58]) were divided into genetic risk factors, parental behaviours (concerning themselves, as well as the child), children’s behaviours, and living conditions.

Genetic risk factors include, for example, gender, age, chronic conditions (of the child and of the parent), (previous) parental illness, premature birth, and birth weight. All of those were assessed, asking parents to state illness, the birth in week of pregnancy, and birth weight using free text.

Parental correlates (concerning themselves) include, e.g., parental overweight or obesity, their screen media use and physical activity behaviour, as well as cigarette smoking and their health consciousness and own body image. All items were assessed for the mother and father separately. BMI was assessed asking for parental height and body weight (so BMI could be calculated), screen media use was assessed on a 7-point scale (0 min to more than 4 h per day), and parental physical activity behaviour was assessed by asking about the minutes per week they spend in different sporting and physical activities. Smoking patterns were assessed by asking whether the mother or father smoked (currently or previously), and healthy consciousness was assessed using a 4-point scale (not at all, a little, very, very much).

Parental behaviours (concerning the child) include, amongst others, breastfeeding the child, smoking during pregnancy, the parental perceived body image of the child (e.g., thinking the child is too fat/thin), and admonishing the child about their weight. The latter two aspects were assessed on a 5-point scale, whereas breastfeeding and smoking during pregnancy were yes/no questions.

Children’s correlates include engagement in physical activity, including active travel to school, their screen media use, and nutrition patterns. Physical activity, for instance, was assessed using an 8-point scale (0 to 7 days) on which parents could rate their children’s daily physical activity as moderate to vigorous in intensity (any activity that increases the heart rate and makes the child out of breath some of the time). Screen media use was assessed for weekends and weekdays separately and parents could estimate how many minutes their child uses screen media per day on a 7-point scale (0 min to more than 4 h). Total screen media use was calculated using a 5:2 ratio for weekdays and weekend days. Soft drink consumption was assessed for at school and out of school with a 6-point scale on how many times soft drinks were consumed (never to more than once per day). Breakfast habits as an indicator for nutrition behaviour were assessed using a 4-point scale asking whether the child eats breakfast in the morning (never, rarely, often, always). Living conditions include maternal and paternal education level, their net household income, their migration background, as well as whether the child is being raised by a single parent. Parental education level (the highest of both parents or the level of the single parent) was dichotomised as a primary and secondary education level vs. a tertiary education level (i.e., high school education vs. no high school education). Monthly household income was assessed using seven categories (below 1250 EUR to 5000 EUR or more) and subsequently dichotomised as low income (below 1750 EUR per month) and medium and high income (1750 EUR or more). Children were classified as having a migration background if at least one parent was born abroad, or if the child was spoken to in a language other than German in the first three years of life.

### 2.4. Data Management and Statistics

Most questions used a 4-, 5-, or 7-point Likert scale, all others were yes/no questions or ones with only two possible answers (e.g., gender). All correlates were dichotomised using a median split and yes or no, respectively. Children’s body weight was dichotomised by overweight/obesity (above the 90th BMI percentile) and normal weight/underweight (90th BMI percentile and below). Migration status was determined regarding whether at least one parent was born outside of Germany, or whether the child had been spoken to in a foreign language during their first years of life. Screen media use and time engaging in physical activity were dichotomised by more or less than 60 min daily. An active commute to school was dichotomised by at least 3 days/week.

Parental body weight and education status were dichotomised by a BMI below or above 25 and an elementary and intermediate education level or a tertiary level, respectively, for the mother and father each. Monthly household income was dichotomised by more or less than 1750 €.

Descriptive statistics including means, percentages, and standard deviations (SD) were calculated. Logistic regression was used to determine odds ratios (OR) for parental and child-related correlates. In order to generate dichotomous variables, the median split or literature-based cut-off values were used to investigate the association with obesity. Missing values are reported in the table in Section 3.1; for all regression models, only cases with existing data were used. The dependent variable was always the child being overweight, whereas independent variables varied. All regression models were adjusted for gender, age, migration background, and family education level. Box–Tidwell tests were used to evaluate logistic regression assumptions. Multiple regression models were performed using those variables (of each part, i.e., child behaviour, parental behaviour, etc.) which became significant in single regression models. All analyses were performed with SPSS Statistics 25 (SPSS Inc., Chicago, IL, USA) using a two-sided significance level of α = 0.05.

## 3. Results

### 3.1. Participants’ Characteristics

Sample characteristics are presented in Table 1. Data of 1956 children were available, of which 1000 (51.1%) were boys and 956 (48.9%) were girls. A total of 190 (10.0%) children were classed as overweight and/or obese.

### 3.2. Genetic Risk Factors Correlating with Childhood Overweight

Logistic regression model analysis for genetic risk factors showed that a diagnosed physical illness of the father and a diagnosed chronic illness of the child are significantly associated with the child being overweight and/or obese (Table 2).

### 3.3. Parental Health Behaviours Correlating with Childhood Overweight

Children being overweight and/or obese was significantly associated with the mother and father being overweight and/or obese. Other behavioural parameters showed no association (Table 3).

### 3.4. Parental Behaviours Peri and Post Pregnancy, Parental Perceptions, and Parenting Practises Correlating with Childhood Overweight

The risk of a child becoming overweight or obese is significantly increased if the child has not been breastfed, if the parents think the child is too fat, and if the mother admonishes the child about their weight (Table 4).

### 3.5. Children’s Health Behaviours Correlating with Childhood Overweight

Engaging in moderate to vigorous physical activity for ≥1 h per day on fewer than 4 days per week was significantly associated with the child being overweight and/or obese (*p* = 0.013). Other behavioural parameters showed no association (Table 5).

In further single logistic regressions, the following results could be derived: Breakfast skipping was shown to be associated with a child being overweight and/or obese. If the child skips breakfast, their risk of being overweight and/or obese is significantly higher than that of a child who frequently or always eats breakfast (OR: 1.778 [1.134; 2.788] *p* = 0.012; controlled for gender, age, and migration background).

For children with no migration background, the risk of being overweight and/obese is increased if the child consumes sweetened beverages more than once a week (OR = 1.743 [1.054; 2.882] *p* = 0.030; controlled for age and gender).

Passive transport to school three times per week or more is also significantly associated with children being overweight and/or obese (OR: 1.481 [1.034; 2.120] *p* = 0.032; controlled for age, gender, and migration background).

Moreover, there is an increased risk of childhood overweight and/or obesity if the child plays games on consoles or computers for ≥1 h per day on weekends (OR = 1.857 [1.026; 3.360] *p* = 0.041; controlled for age, gender, and family education level).

### 3.6. Child’s Living Conditions Correlating with Childhood Overweight

If the child’s mother is currently unemployed or looking for work, the child’s risk of being overweight and/or obese is significantly increased (Table 6).

Further, if analysed in single logistic regression models, if the mother was younger than 30.7 years old when the child was born, the risk of the child being overweight and/or obese is significantly higher than that for children whose mothers are older than or equal to 30.7 years old at the time of the birth of their child (controlled for the gender and age of the child; OR: 1.549 [1.091; 2.198] *p* = 0.014).

Also, if the child was older than 35 months before they went to external care regularly (more than 2 days a week), the child had 1.677 higher odds of becoming overweight and/or obese ([1.091; 2.578] *p* = 0.018; controlled for age and gender).

Growing up with a single parent increased the risk of the child being overweight and/or obese significantly (OR = 1.907 [1.205; 3.019] *p* = 0.006; controlled for age and gender). And, if the child’s mother uses screen media for ≥2 h per day, the child’s risk of being overweight and/or obese is significantly increased (OR: 1.582 [1.036; 2.415] *p* = 0.034; controlled for age and gender).

## 4. Discussion

In this study, a multiplicity of independent factors has been considered and adjusted for. Results showed that apart from non-changeable determinants, such as children’s genetics and surroundings, primarily variable lifestyle factors, parental perceptions, and parenting practises can be associated with an increased risk of childhood overweight. Therefore, those changeable factors need to be addressed in primary prevention measures.

### 4.1. Genetic Risk Factors Correlating with Childhood Overweight

One of the investigated factors was the children’s gender. These data indicate that boys are more often affected by being overweight and/or obese than girls (10.5% and 9.5%, respectively). However, in this study, there is no significant association between gender and being overweight (*p* = 0.478). A nationwide survey presented similar figures [4] and a study examining 636,933 European children between the ages of 6 and 9 years also showed that boys are more likely to be overweight than girls [59,60]. The trend that men are more likely to be overweight than women is also reflected among adults [60,61], which is also seen here: 31.4% of mothers and 60.9% of fathers were overweight and/or obese.

In addition to gender, age was shown to be a significant correlate for children being overweight and obese. This study indicates that children aged 7 years old and older have a higher risk of being overweight or obese, which aligns with findings in some national surveys [4]. However, this contrasts with results from a large European sample where no such association was found [59]. This difference may suggest the influence of regional or cultural factors in the relationship between age and obesity risk in children.

Further, the association between paternal physical illness (such as hypertension, diabetes, and cardiovascular diseases [60]) and children being overweight is highlighted. While it has been established that overweight parents (especially mothers) are linked to higher rates of overweight children [62], the specific link between a father’s chronic illness and the child’s weight status is less commonly discussed. This study brings attention to this previously underexplored factor, which is significant because it expands the scope of familial and environmental factors influencing childhood obesity. This study also emphasises the relationship between children’s chronic illnesses (e.g., asthma, diabetes, ADHD) and obesity. While such associations have been reported previously [17,18], the inclusion of chronic illness as a key factor in shaping childhood obesity risk is noteworthy for highlighting the broader scope of health conditions that contribute to children being overweight. This suggests a need for integrated health approaches that address both chronic illnesses and weight management. These aspects of the study point to the importance of considering a broad range of factors, particularly paternal health and chronic conditions, when addressing childhood obesity. The study’s novelty lies in its comprehensive look at familial and health-related predictors, which may help shape future interventions aimed at reducing childhood obesity risk.

### 4.2. Parental Health Behaviours Correlating with Childhood Overweight

Parents can affect their children’s behaviour and thus the risk of being overweight and obese in childhood, both via their own behaviour, acting as role models, and through educational measures and their parenting style [28,29].

In the present data set, childhood overweight and/or obesity was positively associated with parental overweight. Several studies have supported this [24,63], and explain the relationship with genetic aspects as well as the child’s eating and physical activity behaviours, which are affected both by parental eating and physical activity behaviours and by parental educational measures, and nutritional or exercise strategies. Here, the parental physical activity was associated with the children’s physical activity, which has been shown to have the potential to reduce the risk of obesity in childhood [64]. This study found that paternal physical inactivity was significantly associated with childhood overweight and obesity, which contrasts with existing research that primarily links maternal inactivity with child inactivity [39]. This suggests that paternal behaviours may play a unique or previously under-explored role in childhood obesity risk.

Yet, other health behaviours, such as parental smoking and parental screen media use, were considered. In the data available here, a significant relationship was found between the parents’ current cigarette smoking behaviour and being overweight and/or obese, which confirms previous results [43]. This study adds nuance to the established relationship between parental smoking and childhood obesity by proposing mechanisms based on tobacco exposure. It highlights how maternal smoking, whether through inhalation or breast milk, might alter metabolic functions in children, which could contribute to obesity later in life [65]. This biological explanation is supported by animal studies, expanding on prior human-centric research [66].

With regard to parental screen media use, these data emphasise the role of maternal screen media use (more than 2 h per day) in contributing to childhood overweight. It also ties in findings about the intergenerational pattern of screen media use, noting that children of parents who use screens excessively tend to have higher screen time themselves [67]. This finding underscores the indirect effects of parental screen behaviours on children’s health habits [41,68].

Not only was parental behaviour assessed in this study, but also the image of their own body. The relationship between the parents’ own body image and the children being overweight is a unique focus. This study found that parents who considered themselves to be “too fat” (based on subjective BMI assessments) were more likely to have children who become overweight or obese. This suggests that the parental perception of body image, not just their actual weight, might affect the children’s health outcomes [69]. We would like to highlight the importance of parental role modelling in shaping children’s behaviours. While this idea is not new, this study’s findings offer specific insights into how changes in parental health behaviours—such as increasing physical activity or reducing smoking—might help mitigate childhood obesity risk.

Parental behaviours peri and post pregnancy, parental perceptions, and parenting practises correlated with childhood overweight. This study builds on existing research by linking maternal smoking during pregnancy to childhood obesity through a potential epigenetic mechanism [70]. It suggests that smoking during pregnancy may lead to epigenetic modifications in the foetal DNA, which could contribute to metabolic dysregulation and obesity later in life [70,71]. This perspective adds depth to the conventional understanding of smoking as a lifestyle risk factor, proposing a biological pathway (epigenetics) that might explain long-term health outcomes like obesity [72,73].

While the association between breastfeeding and the lower risk of childhood obesity is well-established, the results emphasise post-prandial insulin secretion differences between formula feeding and breastfeeding [74,75]. Specifically, it suggests that higher insulin secretion after formula feeding could contribute to obesity risk [76,77]. This offers a mechanistic explanation that connects infant feeding practises with long-term health outcomes, highlighting how subtle metabolic differences could affect childhood growth patterns [78].

This study also explored the novel idea that parents’ perceptions of their child’s weight—whether they view their child as “somewhat fat” or “very fat”—are linked to the child’s actual overweight or obesity status [79]. While this may seem intuitive, the text further delves into how the misjudgement or denial of a child’s overweight status might affect parenting behaviours, making it harder for parents to adopt healthy interventions [80]. This focus on parental self-reflection adds complexity to the understanding of how psychological factors influence childhood obesity.

In addition, this study introduces a connection between parental admonishment about a child’s weight and an increased risk of obesity. It points out that children who are admonished for their weight by parents are at a higher risk of being overweight or obese, with gender differences (with girls being more likely to be admonished) further complicating the dynamic. This finding highlights the potentially counterproductive effects of negative weight-focused parenting practises, which may inadvertently encourage unhealthy eating behaviours, such as binge eating, due to stigmatisation and lower self-esteem [81].

Further, restrictive parenting practises—such as imposing dietary limits or focusing too much on a child’s weight—can be associated with the risk of obesity. It has been shown that parents’ concerns about their child’s weight can lead to restrictive eating behaviours, a link that is backed by previous research but emphasised here in the context of how such behaviours may contribute to the child’s eventual weight issues [82,83]. This study touches on the psychological aspects of parenting, revealing how certain interventions may inadvertently backfire.

It can be stressed that parental behaviours, perceptions, and practises connected to childhood obesity are changeable and could be targeted as part of preventive measures, starting as early as during pregnancy. This early intervention focus is novel in that it calls for proactive, early-stage behavioural change in parents, which could be a foundation for tackling childhood obesity from the earliest stages of life.

This study analysed the integration of epigenetic explanations for smoking-related obesity risk, metabolic differences between feeding practises, the psychological effects of weight-related parenting (admonishment, perception, and stigma), and the call for early interventions during pregnancy to address modifiable parental behaviours. These contributions offer new ways of thinking about how parental influences, both biological and psychological, play a significant role in childhood obesity prevention.

Children’s health behaviours correlate with childhood overweight. Apart from parental behaviours, children’s behaviours were also assessed. These include the children’s diet, physical activity behaviour, and screen media use. A key finding is that skipping breakfast was significantly associated with childhood overweight and obesity, which is a relationship that has been confirmed by previous studies, but here, the novelty comes in the indirect mechanism linking breakfast skipping with snacking and poor dietary choices later in the day. Specifically, the results of this study show that children who skip breakfast are more likely to consume unhealthy snacks, like chocolate or chips, in the morning [84]. This reinforces the idea that breakfast skipping could be a gateway behaviour leading to further unhealthy eating habits throughout the day. It can be highlighted that this pattern could be influenced by parental behaviours, as parents may model breakfast skipping either by not offering breakfast or by skipping it themselves [34].

Also, consuming sugar-sweetened beverages more than once a week significantly increases the risk of obesity, particularly in girls and children without a migration background. This highlights a novel demographic insight—that the relationship between sugary drink consumption and childhood obesity may not be uniform across all groups, and that gender and migration background could be associated with how strongly these behaviours are associated with obesity risk [35]. This demographic focus adds depth to the more general associations found in previous research.

One of the most interesting findings is the paradoxical relationship between moderate to vigorous physical activity and obesity risk. The data suggest that children who engage in the recommended amount of physical activity (at least one hour per day) may have an increased risk of being overweight or obese. This contradicts most research, which links physical activity with a lower risk of obesity [36,85]. We hypothesise that this could be due to the higher energy intake of more active children, or because parents of overweight children may overestimate their child’s physical activity levels [86]. This insight is particularly novel because it challenges the assumption that physical activity alone is always protective against obesity and suggests that parental perceptions might play a role in shaping children’s activity levels.

Further, a significant association between excessive screen media use (more than an hour per day) and overweight/obesity was found, which aligns with existing research [67,87]. However, the multidimensional way screen time is associated with childhood obesity is new. Screen media use is not just linked to reduced physical activity but also to an increased consumption of unhealthy foods and a decreased intake of fruits and vegetables [88,89]. Additionally, it can be suggested that screen time is associated with children’s ability to recognise hunger and satiety cues, leading to poor dietary choices [90]. This focus on the psychological and behavioural mechanisms underlying the screen time–obesity link is a more comprehensive take on this well-known association.

Therefore, preventive measures should not only target children’s behaviours directly but should also incorporate family-based strategies to address the parental influence on child health behaviours. This holistic view is novel in that it emphasises the need to improve parental health literacy and parental modelling to support healthier childhood behaviours. It underlines that behaviours like eating breakfast, avoiding sugary drinks, and limiting screen time are not only shaped by the child but are also influenced by parental actions and attitudes, making the family unit a crucial point of intervention for obesity prevention.

### 4.3. Child’s Living Conditions Correlating with Childhood Overweight

Not only are behaviours associated with children’s health and weight statuses, but also non-changeable determinants. In the present data set, a significant association was found between children being overweight and their migration background, which has been shown previously [91] but adds new emphasis onto how this factor can interact with other socio-economic and cultural determinants. The idea that this relationship is culturally determined and associated with socio-economic factors such as family income and parental education adds a more holistic understanding of how a migration background contributes to obesity risk, rather than viewing it as a single, isolated factor.

Socio-economic status (SES), defined by parental education level, household income, and parental employment, was put in connection with the risk of childhood obesity. This demonstrates that children from households with a low or medium parental education level, a low household income (below €1750 monthly), and unemployed mothers face a significantly higher risk of being overweight or obese. This finding underscores the intersectionality of various socio-economic variables, suggesting that the combination of these factors creates a unique and compounded risk profile for childhood obesity.

The study’s emphasis on how socio-economic disadvantage—specifically, low income and unemployment—contributes to childhood obesity provides a more granular perspective on how the broader social and economic context shapes health outcomes. While previous studies have linked socio-economic status to obesity [45,92], this study adds specificity by examining the interplay between multiple socio-economic variables, showing that socio-economic disadvantage leads not only to limited access to healthy foods but also to reduced opportunities for physical activity in socio-economically disadvantaged neighbourhoods [40]. It has been shown that environmental factors, such as the availability of energy-dense foods, disproportionately affect children from lower socio-economic backgrounds [89]. Children in these neighbourhoods consume more unhealthy foods (e.g., energy-dense snacks and sugary drinks) while engaged in screen media use [89], which adds a layer of understanding about the environmental triggers for obesity, which is a growing area of interest in public health research.

This study extends the usual focus on education by showing that not only parental education but also household income and parental employment status play a role in determining parents’ health literacy and capacity to adopt healthy behaviours. The link between lower educational levels and a lack of knowledge about healthy eating, physical activity, and breastfeeding highlights how health disparities can be perpetuated across generations, particularly when resources (both those that are informational and material) are limited.

Addressing childhood obesity requires not only interventions at the individual or familial level but also systemic change. By identifying socio-economic disparities as key drivers of obesity, those findings underscore the need for public health interventions that tackle both the environmental (e.g., access to healthy foods and safe spaces for physical activity) and educational barriers faced by socio-economically disadvantaged families. This adds a macro-level perspective to the often micro-level interventions that focus on individual behaviours.

Therefore, the novelty of this study lies in its multi-dimensional exploration of the socio-economic determinants of childhood obesity, particularly how the migration background, parental education, household income, and employment status collectively are associated with children’s health outcomes. It presents a holistic view of childhood obesity that incorporates not only individual behaviours but also systemic, socio-economic, and environmental factors, offering new insights for prevention strategies that take these complex variables into account.

### 4.4. Strengths and Limitations

Nonetheless, this study is not without limitations, which need to be considered when interpreting these results. Although, a strength of this study lies in its large sample size in terms of identifying risk factors for childhood obesity. However, since this study was purely observational and only cross-sectional, no causality can be derived from those results. Also, results are transferrable within southwest Germany but cannot be generalised globally, especially when comparing high-income regions and high- and medium-income regions. Limitations include the reliance on self-reported data from the participants with the possibility of a selection and recall bias, as well as a social desirability bias, since most parameters were assessed subjectively, and participation was voluntary. Although the used instruments included validated questions [58], those were issued in German only, which could potentially represent a barrier for parents whose native language is not German. Yet, children’s anthropometric measurements were collected objectively and reproducibly by trained staff.

## 5. Conclusions

This study highlights the significant role of parental health, lifestyle, and social behaviours in childhood obesity. Key risk factors include genetic influences, such as a history of paternal illness or a child’s chronic condition, as well as parental overweight, smoking, physical inactivity, and negative perceptions of their child’s weight. Children’s own behaviours, such as their physical inactivity, screen time, and low socio-economic status (e.g., low income and parental education), were also linked to higher obesity risk. Given the long-term health implications of childhood obesity [60], interventions should target both children and parents to address modifiable risk factors and improve health literacy. However, as this was an observational, cross-sectional study, further research is needed to explore the long-term impact of weight management and health changes over time. Longitudinal studies are recommended to provide deeper insights.

## Figures and Tables

**Table 1:** Participants’ characteristics.

	Missing Values	Total (n = 1956)	Boys (n = 1000)	Girls (n = 956)
Age (years), m (sd)		7.1 (0.6)	7.1 (0.6)	7.1 (0.6)
BMI, m (sd)	63	16.0 (2.2)	16.0 (2.2)	16.0 (2.2)
BMIPCT, m (sd)	63	49.0 (27.9)	48.8 (27.9)	49.2 (27.9)
Overweight/obesity, n (%)	63	190 (10.0)	102 (10.5)	88 (9.5)
Child has been breastfed, n (%)	253	1424 (83.6)	710 (82.6)	714 (84.7)
Child is chronically ill ^a^, n (%)		252 (12.9)	128 (12.8)	124 (13.0)
Migration background ^b^, n (%)	310	525 (31.9)	255 (30.9)	270 (32.9)
Primary/secondary family education level, n (%)	336	1098 (67.8)	558 (68.1)	540 (67.4)
Household income < 1750 €, n (%)	465	1284 (86.1)	653 (86.6)	631 (85.6)
No breakfast skipping, n (%)	249	1484 (86.9)	770 (89.6)	714 (84.2)
Sugar sweetened beverages ≥ 1 time/week, n (%)	288	129 (7.7)	61 (7.3)	68 (8.2)
MVPA ^c^ ≥ 1 h/day on <4 days/week, n (%)	333	437 (26.9)	260 (31.7)	177 (22.1)
Active transport ^d^ to school on ≥3 days/week, n (%)	266	1095 (64.8)	550 (64.6)	545 (65.0)
Screen media use ≥ 1 h/day, n (%)	263	244 (14.4)	135 (15.9)	109 (12.9)
Maternal overweight/obesity ^e^, n (%)	376	496 (31.4)	247 (30.8)	249 (32.0)
Paternal overweight/obesity ^e^, n (%)	481	898 (60.9)	443 (59.1)	455 (62.7)
Single parent, n (%)	278	177 (10.5)	82 (9.7)	95 (11.4)

m = means; sd = standard deviation; BMI = body mass index; BMIPCT = BMI percentiles [54]; (^a^) diagnosed hay fever, neurodermatitis, bronchial asthma, obstructive bronchitis, heart disease, anaemia, epileptic seizures, other cramps, thyroid disease, diabetes mellitus, scoliosis, attention deficit disorder/hyperactivity; (^b^) parent(s) were born abroad or the child predominantly spoke a language other than German in the first few years of life; (^c^) MVPA = moderate to vigorous physical activity; (^d^) travel by foot or bicycle; (^e^) BMI ≥ 25 kg/m^2^.

**Table 2:** Genetic risk factors associated with childhood overweight and/or obesity derived from a multiple logistic regression model (n = 1523).

	OR	*p*	95% CI
Father is physically ill ^a^	1.907	0.009	[1.179; 3.085]
Child is chronically ill ^b^	1.692	0.022	[1.078; 2.656]
Child’s gender (male)	1.091	0.640	[0.758; 1.569]
Young age of the child	1.297	0.163	[0.900; 1.869]
Child’s migration background ^c^	1.530	0.027	[1.050; 2.230]
Primary or secondary family education level	1.998	0.002	[1.276; 3.129]

OR = odds ratio; CI = confidence interval; (^a^) diagnosed hypertension, increased blood lipid levels, diabetes mellitus, metabolic syndrome, thrombosis, coronary heart problems, heart attack, stroke; (^b^) diagnosed hay fever, neurodermatitis, bronchial asthma, obstructive bronchitis, heart disease, anaemia, epileptic seizures, other cramps, thyroid disease, diabetes mellitus, scoliosis, attention deficit disorder/hyperactivity; (^c^) parent(s) were born abroad or the child predominantly spoke a language other than German in the first few years of life.

**Table 3:** Risk factors relating to parental health behaviours associated with childhood overweight and/or obesity derived from a multiple logistic regression model (n = 1282).

	OR	*p*	95% CI
Mother is overweight/obese ^a^	1.900	0.022	[1.096; 3.296]
Father is overweight/obese ^a^	2.165	0.011	[1.192; 3.933]
Mother smokes	1.641	0.071	[0.959; 2.807]
Father smokes	1.458	0.132	[0.893; 2.379]
Mother thinks she is too fat	1.361	0.296	[0.763; 2.430]
Father thinks he is too fat	1.018	0.945	[0.609; 1.703]
Father is inactive	1.263	0.317	[0.799; 1.995]
Child’s gender (male)	1.128	0.592	[0.726; 1.751]
Young age of the child	1.494	0.077	[0.958; 2.330]
Child’s migration background ^b^	1.378	0.182	[0.860; 2.208]
Primary or secondary family education level	1.316	0.309	[0.775; 2.236]

OR = odds ratio; CI = confidence interval; (^a^) BMI ≥ 25 m^2^/kg; (^b^) parent(s) were born abroad or the child predominantly spoke a language other than German in the first few years of life.

**Table 4:** Parental behavioural risk factors peri and post pregnancy, parental perceptions, and parenting practises associated with childhood overweight and/or obesity derived from a multiple logistic regression model (n = 1404).

	OR	*p*	95% CI
Mother smoked during pregnancy	1.502	0.307	[0.688; 3.280]
Child has not been breastfed	2.026	0.031	[1.068; 3.841]
Mother thinks child is too fat	8.836	<0.001	[3.788; 20.613]
Father thinks child is too fat	9.949	<0.001	[3.768; 26.266]
Mother admonishes child about their weight	9.320	<0.001	[3.853; 22.547]
Father admonishes child about their weight	1.582	0.253	[0.721; 3.472]
Child’s gender (male)	0.663	0.138	[0.386; 1.141]
Young age of the child	0.781	0.366	[0.457; 1.335]
Child’s migration background ^a^	0.888	0.679	[0.506; 1.559]
Primary or secondary family education level	1.911	0.054	[0.990; 3.690]

OR = odds ratio; CI = confidence interval; (^a^) parent(s) were born abroad or the child predominantly spoke a language other than German in the first few years of life.

**Table 5:** Children’s health behavioural risk factors associated with childhood overweight and/or obesity derived from a multiple logistic regression model (n = 1427).

	OR	*p*	95% CI
MVPA ^a^ ≥ 1 h/day on <4 days/week	0.605	0.013	[0.407; 0.900]
Screen media use ≥ 1 h/day (on school days)	1.125	0.918	[0.118; 10.753]
Screen media use ≥ 1 h/day (on weekend days)	1.478	0.084	[0.949; 2.301]
Screen media use ≥ 1 h/day (total)	1.412	0.766	[0.145; 13.749]
Child’s gender (male)	1.195	0.356	[0.818; 1.746]
Young age of the child	1.347	0.125	[0.921; 1.969]
Child’s migration background ^b^	1.196	0.385	[0.798; 1.793]
Primary or secondary family education level	1.720	0.022	[1.082; 2.733]

OR = odds ratio; CI = confidence interval; (^a^) MVPA = moderate to vigorous physical activity; (^b^) parent(s) were born abroad or the child predominantly spoke a language other than German in the first few years of life.

**Table 6:** Child’s living conditions associated with childhood overweight and/or obesity derived from a multiple logistic regression model (n = 405).

	OR	*p*	95% CI
Mother is currently unemployed/looking for work	3.829	0.019	[1.246; 11.762]
Household income < 1750 € ^a^	1.702	0.167	[0.801; 3.617]
Child’s gender (male)	1.608	0.152	[0.840; 3.078]
Young age of the child	0.950	0.876	[0.496; 1.818]
Child’s migration background ^b^	2.000	0.045	[1.017; 3.934]
Primary or secondary family education level	2.541	0.035	[1.068; 6.048]

OR = odds ratio; CI = confidence interval; (^a^) average monthly household income (net) that all household members have together after deducting taxes and social security contributions (including parental allowances and child benefits); (^b^) parent(s) were born abroad or the child predominantly spoke a language other than German in the first few years of life.

## Data Availability

The data supporting the conclusions of this article will be made available by the authors on request. The date are not publicly available due to legal reasons.
